# Effect of Diosmin on Selected Parameters of Oxygen Homeostasis

**DOI:** 10.3390/ijms241612917

**Published:** 2023-08-18

**Authors:** Marcin Feldo, Magdalena Wójciak, Sławomir Dresler, Paweł Sowa, Bartosz J. Płachno, Dariusz Samborski, Ireneusz Sowa

**Affiliations:** 1 Department of Vascular Surgery, Medical University of Lublin, Staszica 11 St., 20-081 Lublin, Poland; 2 Department of Analytical Chemistry, Medical University of Lublin, Chodźki 4a, 20-093 Lublin, Poland; magdalena.wojciak@umlub.pl (M.W.); slawomirdresler@umlub.pl (S.D.); 3 Department of Plant Physiology and Biophysics, Institute of Biological Sciences, Maria Curie-Skłodowska University, Akademicka 19, 20-033 Lublin, Poland; 4 Department of Otorhinolaryngology and Oncological Laryngology, Faculty of Medical Sciences in Zabrze, Medical University of Silesia in Katowice, 40-055 Katowice, Poland; psowa@sum.edu.pl; 5 Department of Plant Cytology and Embryology, Institute of Botany, Faculty of Biology, Jagiellonian University in Kraków, 9 Gronostajowa St., 30-387 Cracow, Poland; bartosz.plachno@uj.edu.pl; 6 Department of Conservative Dentistry and Endodontics, Medical University, Chodźki 6, 20-093 Lublin, Poland; dariusz.samborski@umlub.pl

**Keywords:** diosmin, chronic venous insufficiency, hypoxia-inducible factor, angiogenesis, interleukins

## Abstract

Chronic venous disease (CVD) is a condition characterized by functional disturbances in the microcirculation of the superficial and deep veins, affecting up to 30% of the global population. Diosmin, a phlebotropic drug, is commonly used in the treatment of CVD, and its beneficial effects have been described in numerous clinical studies. However, the precise molecular mechanism underlying the activity of diosmin is not yet fully understood. Therefore, the objective of our study was to investigate whether diosmin has an impact on oxygen management, as cardiovascular diseases are often associated with hypoxia. In our study, patients were administered a daily dosage of 2 × 600 mg of diosmin for 3 months, and we evaluated several factors associated with oxygen management, angiogenesis, and inflammation using biochemical assays. Our findings indicate that diosmin reduced the levels of fibroblast growth factors (FGF) and vascular endothelial growth factor (VEGF-C), while increasing endostatin and angiostatin levels, suggesting a potential influence on angiogenesis regulation. Furthermore, diosmin exhibited anti-inflammatory properties by suppressing the levels of tumor necrosis factor-alpha (TNF-α), interleukin 1-beta (IL-1β), and interleukin 6 (IL-6), while promoting the production of interleukin 12 (IL-12). Additionally, diosmin significantly decreased the levels of hypoxia-inducible factor (HIF), anion gap (AG), and lactate, indicating its potential influence on the hypoxia-inducible factor pathway. These findings suggest that diosmin may play a crucial role in modulating oxygen management and inflammation in the context of chronic venous disease.

## 1. Introduction

The circulatory system, also referred to as the cardiovascular system, plays a vital role in maintaining physiological functions in all tissues. It is involved in distributing oxygen and nutrients to living cells, regulating body temperature, and eliminating biological waste products from the organism [1]. Disruption of circulation, which often accompanies various cardiovascular disorders, leads to reduced oxygen delivery, resulting in hypoxia and tissue dysfunction [2].

Chronic venous disease (CVD) is a disorder characterized by functional disturbances in the microcirculation of the superficial and deep veins. Incompetent venous valves result in blood reflux, leading to increased and sustained venous hypertension. This increased hydrostatic pressure affects the superficial subcutaneous veins and capillaries, causing transcapillary filtration and excessive lymphatic flow, which in turn leads to the formation of edema. Edema, skin lesions, and structural changes in the vein wall, such as varicose veins and venous leg ulcers, are the most common symptoms of CVD [3]. The most advanced stage of CVD is referred to as chronic venous insufficiency (CVI). The prevalence of CVD is estimated to be around 10% to 30% of the global population, and there is an observed increasing trend associated with obesity and the aging population. This disorder has a significant impact on the quality of life and poses a substantial economic burden [4,5,6].

Several molecular mechanisms have been associated with the development of chronic venous disorders, including the induction of matrix metalloproteinases (MMPs) and the activation of cytokines [7]. Matrix metalloproteinases (MMPs) are involved in the remodeling of capillary walls. They can degrade components of the extracellular matrix, including collagen and elastin, leading to structural changes in blood vessels. This remodeling can affect the integrity and function of the capillary walls, contributing to the progression of CVD. In turn, cytokines regulate various aspects of the immune response and inflammation. In the context of CVD, the induction of cytokines leads to chronic inflammation within the region of lower limbs.

The treatment options for CVD primarily consist of conservative therapy, or in advanced stages, surgical interventions, and endovenous ablation [8]. However, in addition to these options, venoactive compounds are also utilized for the prevention and alleviation of CVD symptoms. Among them, diosmin, a naturally occurring flavonoid, holds significant importance. Numerous clinical studies have demonstrated its efficacy in CVD management. Diosmin offers several benefits. It improves the elasticity of blood vessels, enhances blood circulation and lymphatic flow, reduces the permeability of blood vessels, and exhibits anti-inflammatory and antiswelling properties [9,10]. It has been evidenced that diosmin significantly improves the quality of life in patients with CVD; however, the biological molecular mechanism underlying the activity of diosmin is not yet fully understood and is the subject of intense investigation. Our study is a continuation of research in this area and the aim of our work was to assess whether diosmin acts through the hypoxia-inducible factor (HIF) pathway.

HIF signaling plays a crucial role in the response to hypoxia, which is often associated with various cardiovascular diseases. HIF promotes vascularization in hypoxic areas and also participates in modulating the inflammatory state [11,12]. In chronic venous disease, the dysregulation of the HIF pathway leads to abnormal expression of angiogenic factors. This dysregulation contributes to the development and maintenance of vascular malformations and also causes pathological remodeling of the vascular wall. Consequently, it contributes to the formation of varicose veins and venous thromboembolism [2,13,14].

## 2. Results

### 2.1. Characteristic of the Participants

Finally, a total of 47 participants were enrolled in the study, with 16 patients belonging to CEAP 2 and 3 and 15 patients in CEAP 4. The mean age of the patients was 44.23 years.

The levels of sodium, potassium, albumin, creatinine, and lipids were within the normal range, indicating the absence of kidney, heart, and liver disorders. The Tiffeneau index (forced expiratory volume in one second % of vital capacity) and left ventricular ejection fraction (LVEF) were found to be within the physiological range. This indicates that patients do not have any abnormalities in their pulmonary function.

No statistically significant differences (*p* ≥ 0.05) were observed among patients from different CEAP groups, except for patient age, where the oldest patients were in the CEAP 4 group.

The essential demographic and clinical information is summarized in Table 1.

### 2.2. Basic Blood Parameters

Basic blood parameters were examinated before and after 3 months of the treatment with diosmin. Generally, all parameters were maintained within the physiological ranges (Figure S1). The values of sodium, potassium, and albumin did not change in a statistically significant manner. However, the level of creatinine was slightly lowered in CEAP 3 after the treatment with diosmin. The lipidogram remained unchanged, except for triglycerides in the CEAP 4 group, which showed a slight decrease after diosmin administration.

### 2.3. Clinical Manifestation

**Figure 1:**
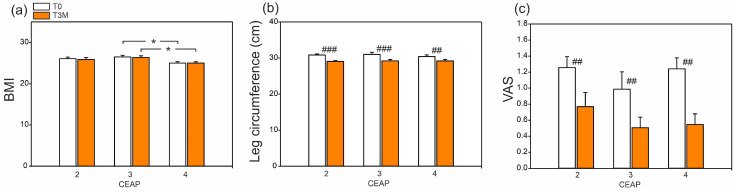
BMI (**a**), leg circumference (cm) (**b**), and pain sensation (**c**) acording to the VAS scale in patients before (T0) and after the three-month treatment with diosmin (T3M). Data are the mean ± SE (*n* = 16 for CEAP 2 and 3; *n* = 15 for CEAP 4). Significant difference between CEAP within the same time are denoted as *p* < 0.05 (*). Significant difference between T0 and T3M within the same CEAP are denoted as *p* < 0.01 (##), *p* < 0.001 (###).

After the three-month treatment with diosmin, a reduction in edema was observed in each group, as evidenced by the decrease in leg circumference. On average, the patients’ leg circumference decreased by approx. 1.7 cm (*p* < 0.001), 1.8 cm (*p* < 0.001), and 1.0 cm (*p* < 0.01), for CEAP 2, 3, and 4, respectively. The sensation of pain was also reduced, and the visual analogue scale (VAS) score decreased by approximately 35% (*p* < 0.001). The BMI of the patients did not change. The data are shown in Figure 1.

In addition, a significant decrease in the incidence of contractions was observed after diosmin therapy (Table 2). Before diosmin treatment, thirty-two participants (68.09%) complained of leg cramps, and after 3 months of diosmin administration, the number decreased to six individuals (12.8%). The decreased was observed in each CEAP groups.

### 2.4. Oxygen Management

**Figure 2:**
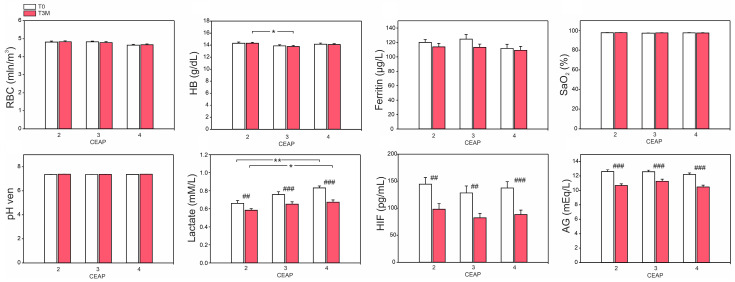
Effect of 3-month diosmin treatment on selected parameters associated with oxygen management: RBC, HB, ferritin, SaO_2_, pH, lactate, HIF, and AG. The data are presented as the mean ± SE with a sample size of *n* = 16 for CEAP 2, 3 and *n* = 15 for CEAP 4. Significant differences between CEAP scores within the same time are denoted as *p* < 0.05 (*), *p* < 0.01 (**). Significant differences between T0 and T3M within the same CEAP are denoted as *p* < 0.01 (##), and *p* < 0.001 (###).

Several parameters involved in oxygen turnover were investigated. Factors associated with oxygen transport, including red blood cell count (RBC), hemoglobin (HB), and ferritin levels (an indicator of iron management), were within the normal range and did not change after diosmin treatment. Saturation (SaO_2_) in all participants was also within the normal range (above 95%), and no differences were observed as a result of the therapy. Considering the potential impact of oxygen delivery disruption on acid–base balance, blood pH, lactate levels, and anion gap (AG) were also investigated in our study. Following diosmin treatment, a statistically significant decrease in lactate levels and anion gap (AG) was observed. Furthermore, a reduction in HIF ranging from 35.1% to 42.4% was noted in each patients’ group. The data are shown in Figure 2.

### 2.5. Markers of Angiogenesis

**Figure 3:**
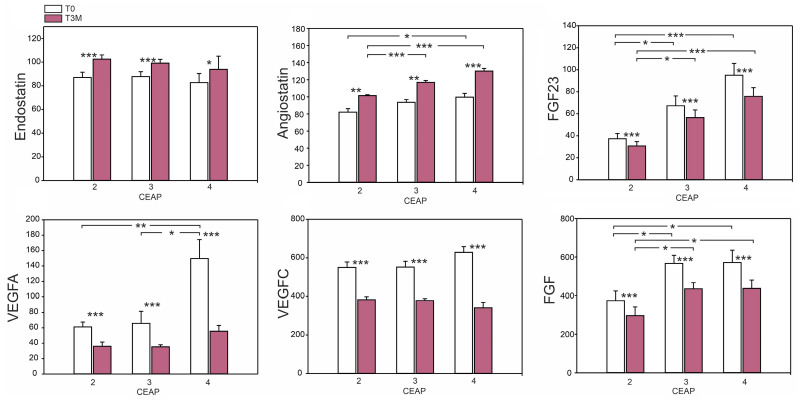
Effect of 3-month diosmin treatment on selected parameters of angiogenesis: endostatin, angiostatin, FGF23, VEGFA, VEGFC, and FGF. The data are presented as the mean ± SE with a sample size of *n* = 16 for CEAP 2, 3 and *n* = 15 for CEAP 4. Significant differences between CEAP scores within the same time are denoted as *p* < 0.05 (*), *p* < 0.01 (**), and *p* < 0.001 (***).

Significant variations were observed in the levels of proangiogenic factors among different CEAP groups prior to diosmin treatment (Figure 3). Fibroblast growth factors (FGF and FGF23) showed significantly higher levels in CEAP 3 and 4 compared to CEAP 2, and VEGF-A was nearly two-fold higher in CEAP 4 than in patients classified as CEAP 2 and 3. However, no differences were observed between groups for the antiangiogenic factors, endostatin and angiostatin.

After three months of diosmin administration, there was a statistically significant decrease in the levels of VEGF-A, VEGF-C, FGF, and FGF23 in all studied groups. Furthermore, the levels of endostatin and angiostatin were elevated compared to the pre-treatment state.

### 2.6. Markers of Inflamation

**Figure 4:**
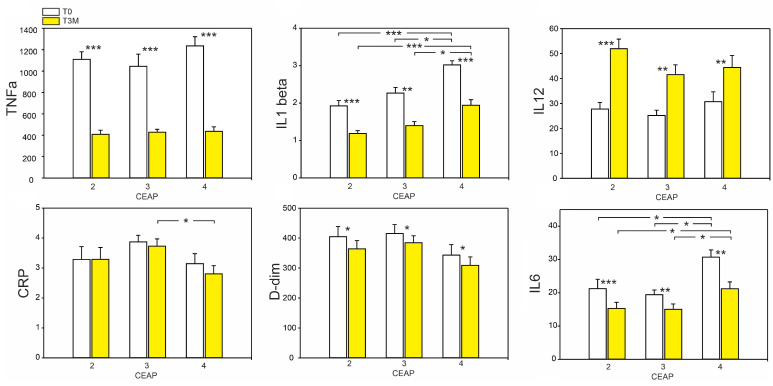
Effect of 3-month diosmin treatment on selected parameters asociated with inflamation: TNFα, IL1β, IL12, CRP, D-dimer, and IL6. The data are presented as the mean ± SE with a sample size of *n* = 16 for CEAP 2, 3 and n = 15 for CEAP 4. Significant differences between CEAP scores within the same time are denoted as *p* < 0.05 (*), *p* < 0.01 (**) and *p* < 0.001 (***).

The administration of diosmin for 3 months resulted in significant changes in parameters associated with the inflammatory state in patients. The mean levels of tumor necrosis factor-alpha (TNF-α), interleukin 1-beta (IL-1β), and interleukin 6 (IL-6) decreased significantly, approximately 3-fold, 1.8-fold, and 1.3-fold lower, respectively, compared to the state before diosmin treatment. On the other hand, the interleukin 12 (IL-12) increased in each group by approximately 1.8-fold. The fibrin degradation product D-dimer also decreased significantly in a statistically significant manner. Furthermore, the levels of IL-1β and IL-6 varied between different CEAP classes and were the highest in CEAP 4 both before and after therapy. No statistically significant differences were observed in C-reactive protein (CRP) content between patients before and after diosmin treatment (Figure 4).

### 2.7. Correlation Analysis

After diosmin treatment a positive correlation was observed between HIF and IL-12 (*p* = 0.021). However, HIF was not correlated with other investigated factors including parameters associated with oxygen management, the other cytokines, as well as factors linked with angiogenesis. Instead, HIF was positively correlated with pain sensation before the therapy (*p* = 0.041). Furthermore, some statistically significant correlation between inflammatory parameters and angiogenetic factors in patients treated with diosmin was found including TNF vs. VEGF A (*p* = 0.000), TNF vs. VEGF C (*p* = 0.028), TNF vs. IL 6 (*p* = 0.017), VEGF A vs. IL6 (*p* = 0.014), VEGF A vs. IL-1β (*p*= 0.002), VEGF-C vs. IL 6 (*p* = 0.013), and IL 6 vs. AG (*p* = 0.024).

The full correlation table is presented in Table S1 (Supplementary Material).

## 3. Discussion

Hypoxia is a pathological condition that is accompanied by the progression of several cardiovascular diseases, among other chronic venous disorders (CVD). It contributes to the formation of various blood vessel pathologies through the activation of hypoxia-inducible factors (HIFs). HIFs mediate cellular and tissue homeostatic responses regulating target genes related to inflammation, vascular remodeling, and angiogenesis, which help in adapting to a low-oxygen environment. In CVD, dysregulation of the HIF pathway leads to alterations in vascular tone, abnormal thrombosis and fibrinolysis, stimulates the expression of angiogenesis factors, and initiates an inflammatory response [2,14,15,16].

According to our previous study, we observed an influence of prolonged diosmin treatment on inflammatory and angiogenic parameters, such as TNF-alpha, VEGF-A, VEGF-C, and FGF-2 [10]. Additionally, some pathways of the regulation of HIF biology by oxygen have been reported in various cells and tissues, including some cancers, atherosclerosis, rheumatoid arthritis, anemia, and stroke [17]. During inflammation, pathological changes in blood flow, such as stasis and edema, along with alterations in vessel integrity and capillary density, lead to impaired tissue oxygenation. It has been postulated that venous hypoxia, caused by blood stasis in patients with chronic venous disease, may contribute to changes in the vein wall [18]. In order to establish a homogenous study group, we excluded patients with cardiovascular and pulmonary comorbidities, smokers, and individuals with anemia or transferrin alterations, as these factors may potentially influence oxidative stress and the hypoxemic state in venous circulation, venous wall, and local tissues hypoperfusion. Prolonged exposure to varicose-vein-dependent blood stasis is believed to lead to venous wall hypoxia due to a reduction in the rate of luminal blood oxygen replenishment compared to normal venous flow. In this pathophysiological context, the 3-month administration of diosmin resulted in reduced blood stasis and improved tissue oxygenation, leading to a decrease in anion gap and serum lactate levels. The parallel decline in serum HIF-1alpha level suggests an improvement in tissue oxygenation. The expression of HIF-1alpha in many tissues increases exponentially as oxygen concentration declines, while its stability and transcriptional activity are negatively regulated by oxygen-dependent hydroxylation [19,20]. Apart from tissue oxygen alteration, the role of HIF as a potential factor linking increased venous pressure and MMPs expression in varicose veins has been supported by several reports. Increased levels and activity of HIF protein in response to mechanical stretch have been observed in various tissues or cell types, including myocardium, fibroblasts, and endothelial cells from capillaries of muscle fibers [21,22]. Prolonged vein wall stretch and wall tension were associated with an increase in HIF-1alpha and MMP-2 and -9 levels and activity, as well as reduced contraction in rat inferior vena cava [23]. The results of our present and previous studies have shown a significant reduction in leg edema, along with a noticeable decrease in HIF-1alpha and MMP-9, cathepsin-L, and endostatin plasma levels at the end of the specific period of diosmin administration [24]. The proinflammatory mediators synthesized at the site of injury, such as TNF-alpha, IL-6, and IL-1beta, further contribute to the expansion of the inflammatory response at the systemic level. Previous studies have revealed a strong association between hypoxia and vein thrombosis, with a confirmed role of the hypoxia-HIF-1alpha-NLRP3-IL-1beta axis as a pathway promoting thrombogenesis. In this pathway, the mRNA expression of IL-1beta was found to be significantly higher in the hypoxia-induced thrombotic group [25]. IL-1beta, IL-6, and IL-12 are recognized as proinflammatory cytokines. Our study demonstrated that a 3-month administration of diosmin led to a decrease in serum activity of IL-6 and IL-1beta, but an increase in IL-12 serum activity was observed. IL-6 and IL-1beta have been established to play crucial roles as acute phase reactants and in innate resistance to various inflammatory challenges [26,27]. Based on our recent observations [10,24], prolonged diosmin administration leads to a decrease in inflammatory factors and proteolysis mechanisms associated with the pathogenesis of cardiovascular disease (CVD). This treatment modulates the intensity of inflammatory processes, leading to a reduction in acute-phase cytokine levels and highlighting the role of IL-12 in the chronic phase of the disease. The observation suggests that the expression of cytokines differs between the acute phase and diosmin-induced remission, making them potential biomarkers for different disease phases or monitoring treatment efficacy.

The members of the IL-12 family (IL-12, IL-23, IL-27, and IL-35) are involved in various biological effects, including inflammatory responses, oxidative stress, and apoptosis. The regulation of inflammation and immune cell differentiation is the most important mechanism for the involvement of IL-12 in the development of cardiovascular diseases [28]. The results of our study showed a significant increase in IL-12 serum levels and a simultaneous decrease in the levels of angiogenic factors such as VEGF and FGF-2 in patients at the end of our study period. Available evidence suggests that this cytokine is involved in the inhibition of angiogenesis by reducing VEGF production in tumor and endothelial cells. Moreover, IL-12 administration reduced the activity of metalloproteinases, which play a role in matrix remodeling [29,30]. In several pathological processes, including cardiovascular diseases (CVD), leukocyte–endothelial interactions play a role in chronic inflammatory conditions. IL-12 activates an antiangiogenic program mediated by INF-gamma in lymphocytes, leading to the downregulation of VEGF in neoplastic and endothelial cells [31]. In the context of angiogenic regulation, we observed a significant increase in antiangiogenic factors, angiostatin and endostatin. The interplay between the two antiangiogenic pathways, i.e., IL-12-angiostatin and IL-12--endostatin, in our observations appears to be influenced by diminished HIF-1alpha activity and improved tissue oxygen balance. On the other hand, the serum level of IL-12 in cardiovascular diseases, observed in arterial and myocardial pathology, showed higher amounts compared to the healthy control [32].

Clinical studies in CVD have reported a positive association between elevations in proinflammatory cytokines such as TNF-alpha, IL-6, and angiogenic factors—VEGF and FGF2 [10]. The above-mentioned IL-1beta, as a potent mediator of the inflammatory response, acts in relation to HIF-1alpha and FGF23 in acute and chronic inflammatory conditions associated with serum phosphates and iron alterations, mainly in chronic kidney disease [33]. Both during and at the end of our study period, in all participants, no alterations in renal parameters (creatinine, urea, and GFR), erythrocytes, phosphates, vitamin D, calcium, iron, and ferritin serum levels were observed. Despite these clinical observations, after 3 months of diosmin administration a significant decrease in FGF23 was noted. A variety of cytokines, including IL-6 and IL-1beta, are released as a consequence of acute inflammation, altering bone and mineral metabolism. However, chronic inflammatory conditions are associated with bone loss [34]. As we consider CVD as chronic inflammation, we could also reveal skin alterations due to hemosiderin deposition [35]. In our study groups, no hemosiderin deposition was seen in the skin of C2 and C3 patients, but it was always present in lipodermatosclerotic skin in the C4 stage of CVD. Notably, the highest FGF23 serum levels were observed in the C4 patient group both at T0 and T3m (Figure 3). Collectively, this data suggests that the FGF23 decrease observed at the end of the study period was not correlated with iron and phosphate serum levels, as revealed in several studies [36,37]. David et al. have shown that the treatment with μg/mL of IL-1beta increases FGF23 mRNA levels in osteoblasts and bone marrow stromal cell culture, suggesting that IL-1beta directly increases FGF23 production independently of systemic iron changes. The increase in FGF23 expression was accompanied by increased cellular expression of HIF-1alpha mRNA and nuclear HIF-1alpha abundance [38]. Thus, in the course of CVD, FGF23 alterations appear related to an intensely proinflammatory cytokine stream.

Altogether, these proinflammatory cytokines accelerate and contribute to adverse outcomes in C2, C3, and C4 CVD, but prolonged diosmin administration changes the strategic directions in the pathophysiology of silencing tissues hypoxia and inflammation. This pathophysiology of inflammation differs between clinical stages, but a low-grade perpetual inflammatory status, clinically defined by normal CRP, could be established as a main feature of CVD. The crucial factor for the development of future therapeutics is understanding the role of hallmark features and revealing new signal transduction pathways, particularly the specific etiology of CVD-related inflammation. However, further studies are necessary to investigate the processing events of CVD following conditions of inflammation and cytokine IL-12 activities with the perspective of new therapeutic agents.

Study limitation: The number of patients involved in our study was not extensive; however, we implemented strict recruitment criteria to ensure the homogeneity of both the control and study groups.

## 4. Materials and Methods

### 4.1. Selection of Patients

The study included a total of 51 individuals (24 males and 27 females) diagnosed with primary chronic venous disease (CVD), who were patients at the Department of Vascular Surgery and Angiology, Medical University of Lublin, between 2016 and 2018. The research protocol was approved by the Independent Ethics Committee of the Medical University of Lublin (consent no. KE-0254/341/2015), and written informed consent was obtained from all participants. The classification of patients was based on Echo-Doppler examination of the leg venous system using a Toshiba Aplio 500 echoscanner (Canon Medical System Europe) with color-flow Doppler and a 5 MHz linear transducer. The patients were classified according to the clinical–etiology–anatomy–pathophysiology (CEAP) classification. The inclusion criterion was the presence of CVD classified as C2, C3, or C4 in the CEAP classification, along with bilateral incompetent greater saphenous veins (GSV) in the femoral and calf regions classified as primary (Ep). Exclusion criteria encompassed conditions such as diabetes, autoimmune disorders, tumors, impaired kidney function, liver disease, recent surgical procedures or trauma, previous vein surgeries, deep/superficial vein thrombosis, congestive heart failure (all patients included had LVFE > 60%), obstructive and restrictive lung disease (all patients included had Tiffeneaux index > 76%), pregnancy, and acute viral infections. Additionally, none of the patients had received diuretics or vasoactive medications for at least three months prior to the recruitment date, and they declared not to have used diuretics or undergone compression therapy during the study period or to have been exposed to high altitude hypoxia and hyperbaric oxygen therapy. Out of the initial group of 51 patients, 4 individuals did not complete the therapy for reasons that remain unknown. As a result, they were excluded from the study.

### 4.2. Diosmin Administration

The patients were administered a daily dosage of 2 × 600 mg of diosmin (Phlebodia, Laboratoires Innothera, Arcueil, France) as was recommended by the drug manufacturer. They were advised to maintain their regular daily habits, including their diet and daily work and rest schedule. Checkup visits were scheduled every 30 days. All assessments were conducted prior to the start of the therapy (T0) and after three months (T3m) of treatment. The patients reported no side effects following the administration of diosmin.

### 4.3. Oedema and Pain Assessment

Edema was evaluated by measuring the circumference of both legs using a tape fixed in the ankle region, positioned 8 cm above the central point of the lateral malleolus, and 13 cm from the floor. This specific positioning was chosen to enhance the measurement’s reproducibility. The procedure was conducted by two independent observers in identical temperature conditions and at the same time of day, precisely 10:00 a.m.

Pain levels were evaluated using a visual analogue scale (VAS). The scale ranged from 0 cm, indicating no pain, to 10 cm, representing intolerable pain.

### 4.4. Blood Collection and Biochemistry Assay

Blood samples were collected in tubes (Sarstedt Monovette EDTA KE, Nümbrecht, Germany) containing EDTA (1 mg/mL) and immediately centrifuged at 1500× *g* for 10 min at 4 °C (Eppendorf centrifuge 5702). Supernatant plasma was separated and stored at –20 °C until the measurement.

The levels of markers were quantified using an enzyme-linked immunosorbent assay kit (ELISA). The experimental procedures were conducted following the instructions provided by the manufacturer.

### 4.5. Anion Gap Assessment

The anion gap analyses were conducted twice, at two time points: T0 and T3m. The methodology used for calculating the anion gap was corrected for albumin [39].

### 4.6. Pulse Oximetry Assessment

The procedure was performed twice, at two time points: T0 and T3m, during 5 min sessions at a stable room temperature of 22 °C. Two devices were placed on the index or middle finger (without nail polish) of both hands. The results were recorded, and the mean arterial oxygen saturation (SaO_2_) was calculated for each session. The blood oxygen saturation (SaO_2_) was assessed using a fingertip pulse oximeter (Mindray SIHC, Hamburg, Germany) following the manufacturer’s instructions and standardized methods [40].

### 4.7. Echo-Doppler Examination

Among the participants, duplex ultrasound was used with standardized methods to provide information on the degree and level of valvular insufficiency (reflux time) and flow velocity. The ultrasound examination was conducted using the Toshiba Aplio 500 echoscanner (Canon Medical System Europe) with a 7–10 MHz transducer. The patients were classified according to the detailed CEAP: Anatomic classification—As (indicating the great saphenous vein) 2 and 3; Pathophysiologic classification—Pr (indicating reflux). During the examination, compression of the vein distally to the probe location showed a spike in venous flow velocity as the blood was pushed anterograde, toward the heart. Valvular insufficiency was defined as the augmentation of Valsalva reflux duration for one second in a standing position [41].

### 4.8. Statistical Analysis

The normality of the data and homogeneity of variance were checked using the Shapiro–Wilk test and Leven’s test (α = 0.05), respectively. The repeated measures analysis of variance (ANOVA) with the time sampling (T0 and T3M) within the same CEAP was used. Additionally, the differences between the CEAP within the same terms of sampling were evaluated using Tukey’s HSD post hoc test. The relationships between variables were estimated using Pearson’s correlation coefficients (*p* < 0.05). Statistical analysis were performed using Statistica software (Tibco Software Inc., Palo Alto, CA, USA).

## Figures and Tables

**Table 1:** The demographic and clinical information of patients involved in the study.

	Total	CEAP			*p*-Value
		2	3	4	
Number of Patients (%)	47 (100)	16 (34.04)	16 (34.04)	15 (31.91)	
Sex, male (%)	24 (51.06)	10 (62.50)	7 (43.75)	7 (46.67)	*p* = 0.523
Mean Age (year)	44.23	42.25	42.60	47.75	*p* = 0.044
Sodium (nmol/L)	44.23 ± 1.02	47.75 ± 1.77	42.25 ± 1.44	42.60 ± 1.85	*p* = 0.044
Potassium (mEq/L)	137.45 ± 0.219	137.31 ± 0.299	137.51 ± 0.412	137.55 ± 0.439	*p* = 0.896
Creatinine (µmol/L)	4.26 ± 0.052	4.24 ± 0.073	4.42 ± 0.101	4.104 ± 0.079	*p* = 0.041
Albumin (g/dL)	70.98 ± 1.52	67.82 ± 2.63	74.69 ± 2.25	70.40 ± 2.88	*p* = 0.171
Cholesterol (mg/dL)	4.49 ± 0.056	4.55 ± 0.099	4.55 ± 0.087	4.39 ± 0.107	*p* = 0.424
LDL (mg/dL)	196.9 ± 1.22	199.4 ± 1.83	197.6 ± 2.38	193.7 ± 1.99	*p* = 0.157
Triglycerides (mg/dL)	101.6 ± 1.64	97.9 ± 2.65	103.7 ± 3.37	103.1 ± 2.29	*p* = 0.289
Index Tiffeneau (%)	113.3 ± 4.25	106.7 ± 6.28	110.5 ± 7.69	123.5 ± 7.69	*p* = 0.252
LVEF (%)	75.74 ± 0.38	75.69 ± 0.55	75.19 ± 0.69	76.40 ± 0.71	*p* = 0.433

LVEF—left ventricle ejection fraction; LDL—low-density lipoprotein.

**Table 2:** The incidence of contractions in patients involved in the study.

	Patients		CEAP						*p*-Value
	(No)		2		3		4		
cramps T0, yes	32 (68.1%)	*p* = 0.000	7 (43.7%)	*p* = 0.028	12 (75.0%)	*p* = 0.003	13 (86.7%)	*p* = 0.016	*p* = 0.029
cramps T3M, yes	6 (12.8%)		1 (6.3%)		1 (6.25%)		4 (26.7%)		0 = 0.148

T0—patients before treatment with diosmin; T3M—after the three-month treatment with diosmin.

## Data Availability

The data presented in this study are available on request from the corresponding author.

## Supplementary Materials

The following supporting information can be downloaded at: https://www.mdpi.com/article/10.3390/ijms241612917/s1.
