# Supplementary material for: Effect of Diosmin on Selected Parameters of Oxygen Homeostasis
Source: Int J Mol Sci. 2023 Aug 18;24(16):12917. doi: 10.3390/ijms241612917 (PMC10454919; doi:10.3390/ijms241612917)
Supplement: Supplementary file 1 [file ijms-24-12917-s001.zip › ijms-2554961-supplementary-4.pdf]

Supplementary material

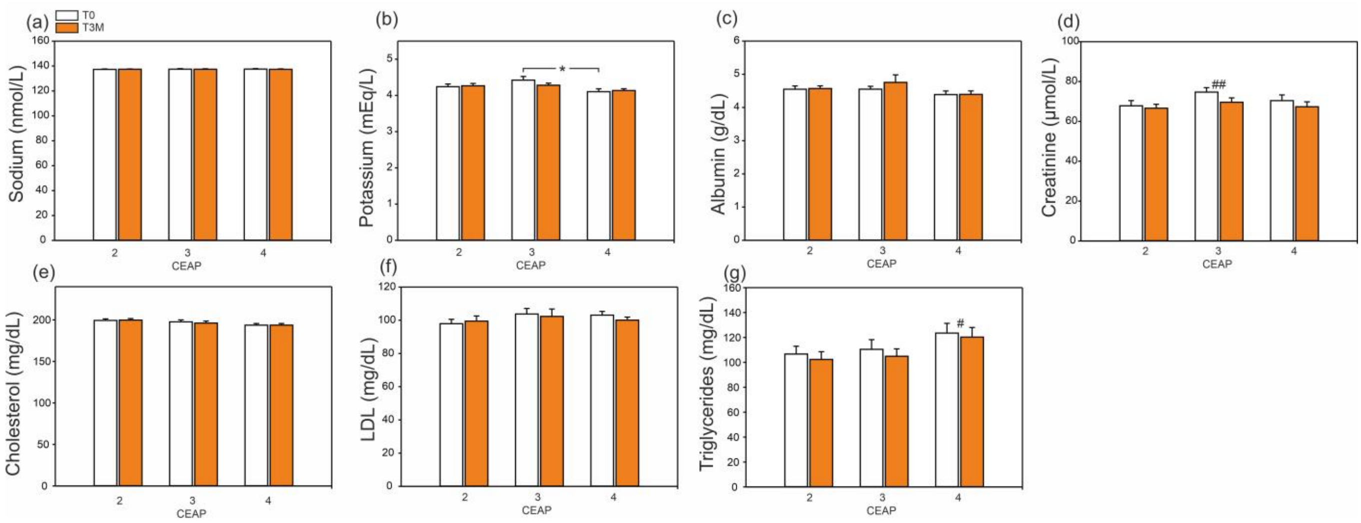

Figure S1. Basic blood paramters in patients before (T0) and after the three-month treatment with diosmin (T3M): (a)- sodium, (b)- potasium, (c)- albumin, (d)- creatinine, (e)- cholesterol, (f)- LDL and (g)- triglycerides. Data are the mean ±SE (n = 16 for CEAP 2 and 3; n = 15 for CEAP 4), significant difference between CEAP within the same time at $p<0.05$ (*), significant difference between T0 and T3M within the same CEAP at $p<0.05$ (#), $p<0.01$ (##).
